# The design and evaluation of a training program on culturally competent psychosocial care provision for men who have sex with men in Senegal

**DOI:** 10.1371/journal.pone.0288018

**Published:** 2023-07-11

**Authors:** Farah Nabil, Kira Grachev, Ángel Gasch-Gallén, Anna Rosés i Belló, El Hadji Mamadou Mbaye, Khady Gueye, Nicole Nkoum

**Affiliations:** 1 Faculty of Health Sciences, University of Zaragoza, Zaragoza, Spain; 2 Department of Psychology, Antioch University, Los Angeles, California, United States of America; 3 The African Women’s Research Observatory, Barcelona, Spain; 4 Social Sciences Department, Institute of Health Research, Epidemiological Surveillance and Training, Dakar, Senegal; 5 Department of Political Sciences, University of Gaston Berger, Saint-Louis, Senegal; 6 La Division de Lutte Contre le SIDA, Ministry of Health and Social Action, Dakar, Senegal; University of Kwazulu-Natal, SOUTH AFRICA

## Abstract

Men who have sex with men (MSM) experience a high degree of discrimination and marginalization in Senegal. Homophobia is pervasive in Senegalese society at the cultural, religious, and political levels. Its effects are reflected in the disproportionately high levels of depression, anxiety, and substance abuse among men who have sex with men when compared to the general population. Given the widespread stigma and the lack of structural support, healthcare providers play a critical role in reconciling the physical and psychological needs of men who have sex with men. This led to the design of a training program that aimed to improve the capacity of healthcare providers to deliver MSM-competent psychosocial care. The training was delivered virtually to 37 Senegal-based nurses and physicians. The program was quantitatively and qualitatively evaluated using pre- and post-testing. The findings demonstrate a general post-training increase in knowledge acquisition (9. 23%, p-value = 0.0021) and a 6.39% reduction in homophobia, which was statistically significant (p = 0.0376); with male providers outperforming female providers, and physicians outperforming nurses. This demonstrates the effectiveness and applicability of the program to support the psychosocial needs of men who have sex with men, and its capacity for future and broader implementation among healthcare providers.

## Introduction

Men who have sex with men’ (MSM) is a term used in this study to describe males who engage in sexual activity with other males, regardless of whether they also have sex with women or have a personal or social gay or bisexual identity. This term is used to inclusively identify men who may or may not identify themselves within the traditional categories of sexual orientation. The population of men who have sex with men in Senegal is highly stigmatized and marginalized, being one of 38 African countries where same-sex activity remains illegal and punishable by a prison sentence [1]. Aside from the state-mandated criminalization of homosexual activity in Senegal, widespread homophobia is largely prevalent in the Senegalese society and is propagated by religious leaders, politicians, and mass media outlets [2]. A recent survey led by the *Réseau national des associations de populations clés* [National network of the associations for key populations] revealed that 84% of the surveyed men who have sex with men in Senegal reported having experienced incidences of stigma, prejudice, and discrimination regularly due to their homosexuality [3]. Such experiences of regular homophobia-induced stigma and discrimination can lead to chronic high levels of stress and anxiety, known as ‘Minority stress’ according to Meyer [4]. In this paper, ’homophobia’ is defined as a range of negative attitudes, feelings, or actions against homosexuality or people who are perceived as being homosexual. This phenomenon demonstrates that marginalized populations, particularly sexual minorities, often experience worse mental and psychosocial health outcomes than their heterosexual counterparts, due to the discriminatory experiences they go through and/or because of a perpetual necessity to conceal their sexuality [4]. Moreover, more data from Senegal and other sub-Saharan African countries such as South Africa, Lesotho, Tanzania, and Kenya reveal that men who have sex with men often bear a disproportionate burden of psychosocial and mental disorders such as depression, anxiety, and substance abuse compared to their heterosexual counterparts [5–9].

In Senegal, despite documented evidence of their experiences of homophobia-induced stigma, discrimination, and chronic stress, there is very little research conducted on their mental and psychosocial health and wellbeing. Outside of the context of HIV and sexually transmitted infections (STIs) prevention, little attention is paid to this so-called ‘key population’. Furthermore, the absence of a national mental health plan in Senegal, makes it more difficult to acknowledge and cater to the mental and psychosocial needs of men who have sex with men.

Previous research revealed that many health providers harbour homophobic attitudes, which has been evidenced to discourage men who have sex with men from interacting with the health system and pursuing health-seeking behaviours in general [10, 11].

Healthcare providers have a crucial role in the process of psychosocial support provision to their clients regardless of their clinical specialty [12]. This may require the competent intervention of a trained provider and some studies have suggested that a state of poor psychosocial health has been documented to worsen health outcomes due to a lower likelihood of adherence to the medical regimen [12]. As per the concept of ‘Whole-person medicine’, psychosocial support is a mandatory part of the healthcare process that all healthcare providers should be familiar with and able to carry out [13]. This approach shifts away from the traditional clinical practice that is strictly biological, and rather moves to a more patient-centered and less disease-centered practice [14]. This process perceives the client as part of a wider context including their family, community, and macro-environment and acknowledges the influence of such factors on the client’s mental, emotional, social, spiritual, and physical health.

Therefore, the primary objective of this training program was to enhance the capacities of healthcare providers in Senegal, mainly nurses and physicians, in providing psychosocial care services tailored for men who have sex with men. The specific objectives of the program were to a) encourage an attitude change where healthcare professionals acknowledge that psychosocial support provision to men who have sex with men is their professional duty, b) increase the healthcare providers awareness and knowledge of the psychosocial health needs and experiences of the men who have sex with men population in Senegal, c) develop the providers’ skills in culturally-competent communication with their clients who are men who have sex with men, and d) strengthen their abilities to provide non-specialized psychosocial support, particularly in the cases of depression, generalized anxiety disorder (GAD) and alcohol use disorder (AUD), to patients who are men who have sex with men and.

## Methods

### Pre-training stage

Prior to the design of this training program, two qualitative research projects were carried out in Dakar, Senegal. The first study, carried out between 2017 and 2019, aimed to explore the psychosocial needs and health-related experiences of men who have sex with men in Senegal [15]. The second study, conducted between 2020 and 2021, aimed to comprehend and analyze the perceptions and attitudes of healthcare providers in Senegal towards men who have sex with men and their psychosocial needs. The findings from these two studies served to inform the design of this training and confirmed its pertinence and need. Thus, the conceptualization of this course took into consideration the psychosocial needs and experiences of men who have sex with men and the gaps in knowledge of healthcare providers in that respect, as well as their need to pursue a homophobia-free clinical practice for their men who have sex with men.

### Recruitment of trainees

The inclusion criteria for potential trainees involved nurses and physicians, currently practicing in any part of Senegal, preferably of a primary healthcare specialty, however, any discipline that involved interaction with male clients was welcome (i.e., obstetricians and gynecologists were not invited to attend the training). The recruitment process was conducted by the Division de la Lutte Contre le SIDA (DLSI) [Division of the fight against AIDS], of the Ministry of Health and Social Action of Senegal. The DLSI has a database of healthcare providers in hospitals around Senegal, which includes their email addresses. This database is used for the purpose of inviting healthcare providers to HIV-related training programs. These potential candidates have then been contacted through email to gauge their interest and availability, and those who have agreed to participate have had their emails shared with the main facilitator of the training. A total of 40 healthcare providers were contacted. They received an official invitation accompanied by the terms of reference. The DLSI managed to successfully recruit 37 candidates that fully attended and completed the training.

### Curriculum

This training program was titled ‘*Prestation de soins psychosociaux compétente pour les HSH (/ F) au Sénégal*’ [Provision of competent psychosocial care for men who have sex with men in Senegal]. The training was conducted fully in French. The program had a duration of 16 hours, excluding the evaluation exercises. It was split into 3 modules. The first module was titled ‘Introduction to psychosocial health’ and its objective was to get the trainees acquainted with the fundamental of psychosocial health and wellbeing from a health and human rights perspective, to gain knowledge on the most commonly prevalent psychosocial disorders on a global scale and in sub-Saharan Africa, and to learn what their role is, as healthcare providers, in the process of psychosocial support. The second module was titled ‘Provision of MSM-competent healthcare’ and focused mainly on increasing the healthcare providers knowledge and awareness of the men who have sex with men population in Senegal, their psychosocial and general health needs, the health obstacles and inequalities they face, and how to incorporate culturally competent communication into their clinical practice and psychosocial care provision for men who have sex with men. The duration allocated to modules 1&2 was 4 hours each. The third and final, titled ‘Psychosocial care for men who have sex with men,’ was allocated 8 hours and primarily focused on the practical aspects of providing psychosocial care. It provided the trainees with hands-on tools to conduct a full psychosocial evaluation and diagnose, manage, and follow up on patients with depression, Generalized Anxiety Disorder (GAD), Alcohol Use Disorder (AUD), and adult malnutrition, and to assess the sexual wellbeing of men who have sex with men. For every 3 hours of theoretical content, an hour of practical group exercises was introduced. The curriculum was developed by the first author of this paper, a global health specialist with expertise in gender, bioethics, HIV, and mental and psychosocial health in vulnerable populations in Africa.

The materials used for the delivery of the training’s content were PowerPoint presentations derived from a Training Manual, authored by the first author of this publication and externally peer-reviewed by 3 experts in the field of psychosocial health of key populations in sub-Saharan Africa. The presentations were complemented by tools from external such as the Marjory Gordon ‘Functional health patterns’ [16], the Generalized Anxiety Disorder-7 scale [17], and the ‘Patient Health Questionnaire-9’ scale [18], which the trainees used to conduct a psychosocial evaluation among other tasks in the third and final module.

This training was designed under the lens of queer bioethics [19] and transcultural healthcare [20], adapted to the Senegalese realities and sociocultural contexts. Wahlert and Feister’s queer bioethics perspective posits that individuals who identify as gay, bisexual, or part of the wider community of men who have sex with men, face the same susceptibility to general health problems and conditions, including chronic non-communicable diseases, as the broader population, and are not exclusively affected by HIV and other sexually transmitted infections. Many training programs, especially in the sub-Saharan African subcontinent, target men who have sex with men exclusively within the sphere of HIV prevention, diagnosis, and treatment. Moreover, the queer bioethics perspective recognizes the distinctiveness of every man who has sex with men, underscoring that their individual needs and experiences might differ, even within the same geographical context. Furthermore, it acknowledges that their identities extend beyond simply being part of a collective of men who have sex with men or other sexual minority groups [21]. Leininger’s transcultural healthcare model, on the other hand, builds its foundation on the concept that a patient’s socio-cultural context will have a substantial impact on the way they perceive, seek, pursue, and experience health, illness, and healthcare. In applying that to the subculture or community of men who have sex with men in Senegal, we find that the vast majority share the collective burden of homophobia-induced stigma and prejudice, which has been evidenced to discourage them from seeking healthcare, especially considering the criminalization of same-sex practices in the country [9]. Adopting these two perspectives, the training program included content that promoted the demarginalization and destigmatization of the men who have sex with men population as a high-risk group that actively propagates HIV in the general population and instead focused on highlighting the structural vulnerabilities that make them more prone to the acquisition of HIV and other sexual and psychosocial diseases that affect this population disproportionately. In addition, the transcultural perspective was enforced through the emphasis on culturally congruent communication with men who have sex with men and the acknowledgment of homophobic experiences, either external or internalized, as a key determinant of psychosocial and overall health and wellbeing.

### Training structure

This training program was originally scheduled to take place between August and September of 2020 in Dakar, Saint-Louis, and Thies, Senegal. However, due to the restrictions implemented to reduce the propagation of the novel coronavirus (COVID-19), the training was conducted online via the Zoom software between September and October 2020. To maximize participation, the 37 trainees were divided into two groups, the first containing 19 and the second group 18 trainees. Each of the two identical training blocks was distributed over 3 days, where each day contained 4 teaching hours. The training was facilitated by the first author of this publication, assisted by a Medical Sociologist based in Dakar, Senegal.

### Evaluation plan

The evaluation materials for this training comprised:

1. Pre- and post-knowledge questionnaires

The pre- and post-test questionnaires were designed to assess the trainees’ knowledge retention and attitude changes immediately upon completion of the training and six months thereafter. The pre-test was administered right before the start of the first training session. The test included 12 questions, corresponding to 1 point each. In conformity with the time allocated to each module, 3 questions were assigned to each of modules 1&2, while module 3 was designated 6 questions. The knowledge questionnaire can be found in the S1 Table.

2. The Homophobia Scale [22]

The Homophobia Scale is a tool designed to measure the degree of homophobic attitudes that an individual fosters through a set of 25 questions, including both normally and reverse-scored items. Normal scoring ranged from 1 (’totally disagree’) to 5 (’totally agree’), while for reverse-scored items, the scale was inverted with 1 as ’totally agree’ and 5 as ’totally disagree’. To ensure consistency in data analysis, all scores were appropriately adjusted. The scale provides its respondents with a set of scenarios that involve a hypothetical interaction with a homosexual individual (e.g., ‘If I discovered a friend was gay, I would end the friendship’) and statements regarding their feelings about homosexuality (e.g., ‘Homosexuality is immoral’, ‘Homosexuality is unacceptable to me’). The three main axes that the scale measures are negative affect, behavioral aggression, and cognitive negativism toward homosexuality.

The homophobia scale was judiciously selected for several reasons. Firstly, this scale has demonstrated its validity and reliability in numerous studies, particularly in the context of Sub-Saharan Africa. Notably, it has been extensively utilized in research papers focused on this region [23–26], thereby establishing a precedence of successful application.

Secondly, we conducted consultations with experts based in Senegal to ensure the cultural appropriateness and relevance of the scale. Their feedback affirmed the suitability of the homophobia scale for our study, taking into consideration the cultural nuances and unique attributes of the population we aimed to study. Such expert validation is critical to ensuring the accuracy of the responses and the overall validity of our research. Finally, we further verified the reliability of our findings by computing Cronbach’s Alpha. Our score of 0.856 reinforces the internal consistency of the homophobia scale in our study context.

The trainees were asked to respond to the Homophobia Scale before the first training session, right after the completion of the training, and 6 months after the training had been completed. The administration of the Homophobia Scale was conducted anonymously using Google Forms. Its administration was allocated 15 minutes. This exercise aimed to assess any changes in homophobic feelings and attitudes of the providers as a result of the training, and to evaluate the sustainability of such a change, if any, 6 months following the training.

In order to assess the homophobia scale’s internal consistency reliability, the Chronbach’s alpha was calculated. Before initiating the training program, the computation was done using the participant responses. The calculation yielded a Chronbach’s alpha of 0.856, which denotes a high degree of internal consistency among the scale’s components. This shows that the scale’s components are assessing the same underlying construct and that participants’ answers to these items are reliable.

3. Satisfaction questionnaires

The satisfaction questionnaire (S1 Table) contained 11 questions with a Likert scale ranging from strongly disagree to strongly agree as possible response options. The questionnaire was designed to measure the trainees’ overall satisfaction with the training, its content, the delivery of the session, the competence of the facilitators, the adequacy of the content regarding the Senegalese context, and the relevance of the training to their clinical practice needs. The questionnaire concluded with an open-answer question asking the trainees to provide suggestions on how this training program could be improved. The questionnaire was administered anonymously using Google Forms and immediately after the training was completed, after which, Cronbach’s Alpha was calculated yielding 0.698 indicating an acceptable level of internal consistency among the questions.

4. Performance indicators

In accordance with an adapted format of Basarab’s Predictive Evaluation Framework [27], the trainees were asked to take part in the evaluation process through post-training self-evaluation and surveillance. On the 3rd and final day of the training, the trainees were asked to participate in an exercise where, in line with the objectives of the program, they were to propose a set of SMART indicators (specific, measurable, achievable, relevant, and time-bound). This exercise was conducted in groups of 4–5 trainees. After the completion of the 2nd training block, the answers of all groups were consolidated, and the duplicates were removed. The choice of the final set of indicators was determined according to the feasibility of each indicator concerning the time frame and the required financial and human resources. Following the training, the trainees were provided with instructions on how to periodically document their progress, or lack thereof, in line with the program objectives. Six months after the completion of the training, each trainee’s progress was evaluated through the indicators and the set of proposed performance indicators. The evaluation took place by means of a Zoom call and the live administration of a structured questionnaire.

### Data analysis

The software Microsoft Excel© was used for data processing and analysis. To analyze the data from the pre- and post-knowledge questionnaires, the following steps were taken. First, a separate analysis of each of the 3 test attempts was conducted through the calculation of descriptive statistics such as mean, median, standard deviation, mode, and range. The scores of each test attempt were then placed side by side and underwent comparative analysis employing a paired and unpaired t-test. A bivariate analysis through paired and unpaired t-tests were run to determine the significant differences in knowledge acquisition based on gender and profession.

Concerning the analysis of the Homophobia Scale results, firstly, the score of each respondent’s scale attempt was computed, denoting their level of homophobia (higher scores indicating higher levels of homophobia). The score could fall anywhere between 0 to 100, where 0 is the lowest level of homophobia and 100 is the highest. The scores resulting from each of the 2 attempts were analyzed individually for descriptive statistics. After which a paired t-test was used to compute the total and disaggregated mean score differences between the first and second attempts.

Descriptive statistics were generated for each of the 11 satisfaction evaluation statements. Each statement could have a score anywhere between 1 and 5, where 1 indicates the highest level of disagreement and 5 indicates a strong agreement with the statement. The overall score for the satisfaction questionnaire was also computed on a scale from 1 to 5. Also, a summary of improvement suggestions was prepared and presented in a narrative form in the Results section.

As for the analysis of the ‘Performance indicators’, data on each performance indicator was collected from the trainees individually 6 months following the completion of the training. The extent to which the achievement of the objective attached to each indicator was measured and converted into a percentage (e.g., Objective: discuss the psychosocial health needs of men who have sex with men during each weekly staff meeting. Indicator: number of staff meetings where the psychosocial health needs of men who have sex with men were discussed. If out of the 24 expected staff meetings (4 meetings*6 months), only 12 meetings included the aforementioned topic of discussion, the score would be (12/24*100 =) 50%). The mean percentage for each indicator was then produced by consolidating the data from all trainees. Qualitative self-assessment and feedback on the performance indicators from the trainees have also been collected and presented below in a narrative format.

### Ethical considerations

The conduct of this training and its evaluation for research purposes underwent appraisal and was approved by the National Health Research Ethics Committee of Senegal (Ref.: SEN18/02). Strict data handling and analysis measures were put in place to ensure the highest degree of privacy and confidentiality for the data of the trainees. Informed consent was sought by means of the invitation letters and terms of reference where information about the study and training were described. The documents stated that by participating in the training and submitting responses to the questionnaires, participants consented to having their responses anonymously processed, analyzed, and reported.

## Results

### Sociodemographic data

The training had 37 participants, comprising 14 women and 23 men (37.84% and 62.16%). As seen in Table 1, physicians represented 59.46% (22) of the participants, while nurses comprised 40.54% (15). Out of the 14 female trainees, 6 were physicians while 8 were nurses, while there were 7 male nurses and 16 male physicians. 70.27% of the trained healthcare providers practiced in Dakar. Most of the physicians were General Practitioners (54.54%). The average age of female trainees was 36.14 years, while for males it was 38.43.

**Table 1:** Sociodemographic data.

		Gender		Total n (%)
		Female n (%)	Male n (%)	
Age	21–30	3 (8.11)	7 (18.92)	10 (27.027)
	31–40	7 (50)	10 (27.03)	17 (45.94)
	41–50	3 (8.11)	1 (2.70)	4 (10.81)
	51–60	1 (2.70)	5 (13.51)	6 (16.22)
Profession	Physicians	6 (16.22)	16 (43.24)	22 (59.46)
	Nurse	8 (21.62)	7 (18.92)	15 (40.54)
Specialty	General practitioner	2 (5.41)	10 (27.03)	12 (32.43)
	Public health	1 (2.70)	1 (2.70)	2 (5.41)
	Infectious diseases	1 (2.70)	-	1 (2.70)
	HIV/STIs	2 (5.41)	-	2 (5.41)
	Addictionology	-	1 (2.70)	1 (2.70)
	Dermatology	-	1 (2.70)	1 (2.70)
	Occupational health	-	1 (2.70)	1 (2.70)
	Urology	-	1 (2.70)	1 (2.70)
	Psychiatry	-	1 (2.70)	1 (2.70)
	N/A (nurse)	8 (21.62)	7 (18.92)	15 (40.54)
Location of Practice	Dakar	11 (29.73)	15 (40.54)	26 (70.27)
	Ziguichor	-	4 (10.81)	4 (10.81)
	Mbao	-	1 (2.70)	1 (2.70)
	Sedhiou	1 (2.70)	2 (5.41)	3 (8.11)
	Louga	1 (2.70)	-	1 (2.70)
	Kaolak	1 (2.70)	-	1 (2.70)
	Thies	-	1 (2.70)	1 (2.70)

### Changes to the degree of homophobia

The Homophobia scale results show that 6 months after the completion of the training program, there was a significant reduction in the mean degree of homophobia of the training participants. As Table 2 shows, the mean Homophobia percentage went from 40.61% to 34.22%. This 6.39% reduction in homophobia was statistically significant (p = 0.0376). The scores on the homophobia scale, after the appropriate transformation of reverse-scored items, ranged from 1 to 5. This diversity in the score range reflects the breadth of attitudes towards homosexuality within our study population.

**Table 2:** Homophobia scale results.

Degree of homophobia (%)		
	Pre-test	Post-test (6 months)
Mean	40.61	34.22
Standard deviation	14.89	10.94
Minimum	7	14
Maximum	75	56
p-value	0.0376	

### Knowledge acquisition

Table 3 shows statistically significant overall knowledge acquisition of 9.23% (p-value = 0.0021) among the 37 participants, according to the pre- and post-training test results. As for the post-6 months’ knowledge level improvement, there was a 5.63% (p-value = 0.04) increase in mean scores in comparison with the baseline.

**Table 3:** Knowledge acquisition.

	Mean score (%)	Standard deviation
Pre-test	4.38 (36.49)	1.78
Post-test	5.49 (45.72)	2.63
Post-6 months test	5.05 (42.12)	1.91
Pre-post test difference	1.11 (9.23), p-value = 0.0021	2.03
Pre-post 6 months test difference	0.67 (5.63), p-value = 0.04	1.93

As seen in Table 4, although female participants performed 0.67% better in the pre-test, they were outperformed by their male counterparts in the post-test and post-6 months test where they showed an 11.23% (p-value = 0.001) and 9.06% (p-value = 0.004) improvement in knowledge level, respectively.

**Table 4:** Sex-disaggregated knowledge acquisition.

	Mean score (%)		Mean difference (%)
	Male	Female	
Pre-test	4.35 (36.23)	4.43 (36.9)	-0.08 (-0.67)
Post-test	5.67 (47.46)	5.14 (42.86)	0.53 (4.6)
Post-6 months test	5.43 (45.29)	4.43 (36.9)	1.0 (8.39)
Pre-post test difference	1.35 (11.23)	0.71 (5.95)	0.64 (5.28)
Pre-post 6 months test difference	1.09 (9.06)	0 (0)	1.09 (9.06)

While nurses and physicians had similar scores in the pre-test, physicians had significantly outperformed the nurses in both the post-test and the post-6 months’ test (p-value = 0.00016, p-value = 0.00017, respectively) as seen in Table 5. In addition, the nurses’ knowledge level seemed to decline after 6 months from the completion of the training (p-value = 0.57).

**Table 5:** Profession-disaggregated knowledge acquisition.

	Mean score (%)		Mean difference (%)
	Physician	Nurse	
Pre-test	4.45 (37.12)	4.27 (35.56)	0.18 (1.5)
Post-test	6.32 (52.67)	4.27 (35.56)	2.05 (17.08)
Post-6 months test	5.82 (48.5)	3.93 (36.9)	1.89 (15.75)
Pre-post test difference	1.86 (15.53)	0 (0)	1.86 (15.53)
Pre-post 6 months test difference	1.36 (11.36)	-0.33 (-2.78)	1.69 (14.08)

### Performance improvement

As seen in Table 6, the participants with the highest targets achieved were in the category of male and female physicians. The performance indicators with the highest achievement rates were indicator 3 “Percentage of MSM clients who underwent a psychosocial evaluation (when deemed necessary)” with an average result of 96%, and the sub-indicators under number 4, related to the diagnosis and treatment of generalized anxiety disorder and depression with an average of 100%. Categories that received a score of N/A indicate that the participants had not had an opportunity to, for instance, administer a test for depression diagnosis due to the absence of patients who were men who have sex with men who exhibited depressive symptoms or due to the absence of men who have sex with men as patients altogether. The performance indicator with the lowest mean score was the “Percentage of male clients who were asked about same-sex practices during a sexual history”, reportedly due to the stigma around same-sex practices, and the healthcare providers’ worry about being harassed by the clients, as they may get offended when asked such a question. The second lowest-scoring performance indicator was the “Percentage of staff meetings where the needs of MSM clients were discussed”. Reportedly, that was due to the perceived lack of relevance of discussions around the health of men who have sex with men by the healthcare providers in leadership positions, who usually steer the conversation during staff meetings. Nevertheless, the training participants still reported trying to encourage discussions around the health of men who have sex with men at least once a month.

**Table 6:** Performance indicators.

Indicator	Female		Male		Total (mean)	Target result
	Nurse (mean)	Physician (mean)	Nurse (mean)	Physician (mean)		
1. Percentage of staff meetings where the needs of MSM clients were discussed	68.75	52.83	46	70.53	59.53	100
2. Number of monthly reflections on one’s interactions with MSM clients	5.3	4	2.75	2.4	3.61	4
3. Percentage of MSM clients who underwent a psychosocial evaluation (when deemed necessary)	88	100	N/A	100	96	100
4.1. Percentage of MSM clients who were assessed for depression using the PHQ-9 (when deemed necessary)	100	N/A	N/A	100	100	100
4.2. Percentage of MSM clients diagnosed with depression who had received adequate care	N/A	N/A	N/A	100	100	100
4.3. Percentage of MSM clients who were assessed for generalized anxiety disorder using the GAD-7 (when deemed necessary)	100	93.33	N/A	70.6	88	100
4.4. Percentage of MSM clients diagnosed with generalized anxiety disorder who had received adequate care	N/A	100	N/A	100	100	100
5. Percentage of male clients who were asked about same-sex practices during a sexual history	38.57	73.75	38.33	62.33	53.26	100

### Trainee satisfaction

The training achieved a generally high satisfaction rate, with a mean overall satisfaction of 4.48 out of 5 (89.5%) across all categories, as seen in Table 7. The statements that received the highest satisfaction rate were: “The lead facilitator mastered the subject” (4.82), “I would recommend this training to my colleagues”, and “The facilitators welcomed the questions and answered them appropriately” (4.66 each). The lowest scoring statement was “The training material was adapted to the context of health service delivery in Senegal” with a rating of 3.87.

**Table 7:** Satisfaction evaluation.

	Mean	Median	Standard deviation
The content of the training met my expectations	4.63	5	0.49
The size of the training group was appropriate	4.5	5	0.65
The training material was adapted to the context of health service delivery in Senegal	3.87	4	1.04
The mix of theoretical and practical content was appropriate	4.29	4	0.77
The lead facilitator mastered the subject	4.82	5	0.46
The facilitators welcomed the questions and answered them appropriately	4.66	5	0.48
The facilitators have conducted and moderated the exercise in an appropriate manner	4.55	5	0.5
The training matched my needs as a healthcare professional	4.61	5	0.55
In general, I am satisfied with the quality of the training	4.55	5	0.55
The training was practical and easy to apply	4.11	4	0.8
I would recommend this training to my colleagues	4.66	5	0.48

## Discussion

Research findings demonstrate a notable disparity in the knowledge acquisition among sexes and professions; with male providers demonstrating greater improvement in knowledge than female providers and physicians outperforming nurses on post-training tests. Previous research that measured perceived homophobia among physicians and nurses found women physicians and nurses to be more homophobic than men [28, 29]. The present research builds on this and corroborates the conclusion that female providers demonstrated poorer knowledge acquisition than their male counterparts. Future research could seek to offer insight as to the significant differences in knowledge acquisition between genders.

Our results reveal that the training program effectively facilitated MSM-competent care for healthcare providers by increasing knowledge acquisition and reducing levels of homophobia. This outcome aligns with previous studies showing that education substantially reduces homophobic discrimination and stigma [28]. Measuring the outcomes of educational training programs such as this one is necessary to advocate for more comprehensive care and to promote future interventions in the healthcare system [30].

The results demonstrate that there was a statistically significant improvement in knowledge acquisition, however; the majority of the trainees scored below 50% in the post-training knowledge tests. This may suggest that the training was not sufficiently attuned to the gap in understanding among healthcare providers, in relation to the psychosocial needs of patients who are men who have sex with men. Perhaps the training program could have been more effectively received if it had been longer in duration and had spanned more days or months. Future training programs might benefit from having vocation-oriented training, particularly as nurses and physicians have considerably different relationships with patients.

This research highlights the considerable improvement in the capacity of healthcare providers to offer MSM-sensitive care. The 5% decline in knowledge acquisition between the pre-training test and 6 months post-training test indicate the effectiveness of the training, however; the pre-training test and immediate post-training test difference compared to the pre-training test and 6-month post-training test difference was 50%. This suggests that knowledge acquisition might hold a temporal component. More consistent training is likely beneficial to support the improvement of psychosocial and medical services offered by healthcare providers.

A study evaluating the attitude shifts after sensitization training of healthcare providers of men who have sex with men in South Africa found that acknowledgment by healthcare providers that men who have sex with men in Africa did not significantly shift after receiving training [31]. This supports the results of the present research that found that the performance indicator with the lowest mean score was the Percentage of male clients who were asked about same-sex practices during a sexual history. This was reportedly related to stigma, and concerns of association with men who have sex with men. Conversely, a study into the attitudes of healthcare providers in Malawi towards men who have sex with men, found that healthcare providers encouraged patients who were men who have sex with men to disclose sexual history [32]. The distinction among study results may be due to several factors, including cultural and religious differences among countries.

The study has important implications for the provision of care to men who have sex with men in Senegal. The results indicate a need for training programs that are better attuned to the specific challenges faced by healthcare providers in this setting, such as stigma and discrimination. In addition to being longer and vocation-specific, future training programs should address the cultural and religious context in which healthcare providers are operating, and seek to foster a supportive and non-judgmental environment. Given the criminalization of homosexuality and the prevalent cultural taboo, it is crucial that healthcare providers are equipped to provide sensitive and effective care to men who have sex with men. Providing such care could help to improve physical and psychosocial health outcomes for this population, which is especially vulnerable to discrimination and poor health.

The generalizability of the study findings is limited due to the small sample size of 37 trainees. While the results of the study suggest that there was a statistically significant improvement in knowledge acquisition, the small sample size raises questions about the representativeness of the findings and their applicability to other populations of healthcare providers. Furthermore, our study did not consider the potential influence of religion on homophobic attitudes, which could be particularly relevant given the observed gender differences in the results. Future research may benefit from incorporating measures of religiosity to explore this dynamic.

In Senegal, men who have sex with men face unabating discrimination and stigma. Given this cultural and structural context, men who have sex with men are more vulnerable to poor physical and psychosocial health than their heterosexual counterparts. The study highlights the importance of culturally and structurally informed training programs for healthcare providers in Senegal to provide better care to patients who are men who have sex with men. The results showed improvement in knowledge acquisition but the majority of trainees still scored below 50%, pointing to the need for more effective training. Disparities in knowledge acquisition and performance indicators between healthcare professions suggest the presence of biases and cultural factors, and the importance of understanding these disparities to develop more equitable and effective training programs. These findings have significant implications for healthcare organizations in Senegal and inform efforts to address health disparities faced by men who have sex with men. A greater understanding of healthcare provider biases could ultimately lead to better care for men who have sex with men.

## Supporting information

S1 Table Knowledge questionnaire. (DOCX)

## References

[1] International Amnesty. Africa: Making love a crime: Criminalization of same-sex conduct in Sub-Saharan Africa [Internet]. 2013. Available from: https://www.amnesty.org/en/wp-content/uploads/2021/06/afr010012013en.pdf

[2] M’Baye B. The origins of Senegalese homophobia: discourses on homosexuals and transgender people in colonial and postcolonial Senegal. Afr stud rev [Internet]. 2013 Sep [cited 2022 Oct 16];56(2):109–28. Available from: https://www.cambridge.org/core/product/identifier/S0002020613000449/type/journal_article

[3] Bangoura D, Coulibaly A S, Laborde-Balen G & Diop A K. Enquête sur l’expérience et les perceptions des HSH âgés de plus de 30 ans, à Dakar, Touba et Mbacké [Internet]. Réseau National des populations clés.; 2018. Available from: https://plateforme-elsa.org/wp-content/uploads/2018/11/RENAPOC-Dakar-EnqueteHSHages2017-2018.pdf

[4] Meyer IH. Prejudice, social stress, and mental health in lesbian, gay, and bisexual populations: Conceptual issues and research evidence. Psychological Bulletin [Internet]. 2003 Sep [cited 2022 Oct 16];129(5):674–97. Available from: http://doi.apa.org/getdoi.cfm?doi=10.1037/0033-2909.129.5.674 12956539 10.1037/0033-2909.129.5.674 PMC2072932

[5] Baral S, Scheibe A, Sullivan P, Trapence G, Lambert A, Bekker LG, et al. Assessing priorities for combination hiv prevention research for men who have sex with men (MSM) in Africa. AIDS Behav [Internet]. 2013 May [cited 2022 Oct 16];17(S1):60–9. Available from: http://link.springer.com/10.1007/s10461-012-0202-5 22610371 10.1007/s10461-012-0202-5

[6] Stahlman S, Grosso A, Ketende S, Sweitzer S, Mothopeng T, Taruberekera N, et al. Depression and social stigma among MSM in Lesotho: implications for HIV and sexually transmitted infection prevention. AIDS Behav [Internet]. 2015 Aug [cited 2022 Oct 16];19(8):1460–9. Available from: http://link.springer.com/10.1007/s10461-015-1094-y 25969182 10.1007/s10461-015-1094-y PMC4858167

[7] Stahlman S, Sanchez TH, Sullivan PS, Ketende S, Lyons C, Charurat ME, et al. The prevalence of sexual behavior stigma affecting gay men and other men who have sex with men across sub-Saharan Africa and in the United States. JMIR Public Health Surveill [Internet]. 2016 Jul 26 [cited 2022 Oct 16];2(2):e35. Available from: http://publichealth.jmir.org/2016/2/e35/ doi: 10.2196/publichealth.5824 27460627 PMC4978863

[8] Niang CI, Tapsoba P, Weiss E, Diagne M, Niang Y, Moreau AM, et al. ‘It’s raining stones’: stigma, violence and HIV vulnerability among men who have sex with men in Dakar, Senegal. Culture, Health & Sexuality [Internet]. 2003 Jan [cited 2022 Oct 16];5(6):499–512. Available from: http://www.tandfonline.com/doi/abs/10.1080/1369105031000152715

[9] Poteat T, Diouf D, Drame FM, Ndaw M, Traore C, Dhaliwal M, et al. HIV risk among MSM in Senegal: a qualitative rapid assessment of the impact of enforcing laws that criminalize same sex practices. Kissinger P, editor. PLoS ONE [Internet]. 2011 Dec 14 [cited 2022 Oct 16];6(12):e28760. Available from: https://dx.plos.org/10.1371/journal.pone.0028760 22194906 10.1371/journal.pone.0028760 PMC3237497

[10] Fay H, Baral SD, Trapence G, Motimedi F, Umar E, Iipinge S, et al. Stigma, health care access, and hiv knowledge among men who have sex with men in Malawi, Namibia, and Botswana. AIDS Behav [Internet]. 2011 Aug [cited 2022 Oct 16];15(6):1088–97. Available from: http://link.springer.com/10.1007/s10461-010-9861-2 21153432 10.1007/s10461-010-9861-2

[11] Magesa DJ, Mtui LJ, Abdul M, Kayange A, Chiduo R, Leshabari MT, et al. Barriers to men who have sex with men attending HIV related health services in Dar es Salaam, Tanzania. Tanzania J Hlth Res [Internet]. 2014 May 14 [cited 2022 Oct 16];16(2). Available from: http://www.ajol.info/index.php/thrb/article/view/94184 doi: 10.4314/thrb.v16i2.8 26875306

[12] Mash R. Whole person medicine: Psychosocial issues in primary care. Afr j prim health care fam med [Internet]. 2016 Nov 16 [cited 2022 Oct 16];8(1). Available from: https://phcfm.org/index.php/phcfm/article/view/1328 doi: 10.4102/phcfm.v8i1.1328 28155311 PMC5125258

[13] Mash R, Almeida M, Wong WCW, Kumar R, von Pressentin KB. The roles and training of primary care doctors: China, India, Brazil and South Africa. Hum Resour Health [Internet]. 2015 Dec [cited 2022 Oct 16];13(1):93, s12960-015-0090–7. Available from: http://www.human-resources-health.com/content/13/1/93 26637305 10.1186/s12960-015-0090-7 PMC4670546

[14] People-Centred Health Care: A policy framework. [Internet]. World Health Organization; 2007. Available from: https://apps.who.int/iris/bitstream/handle/10665/206971/9789290613176_eng.pdf.

[15] Nkoum N, & Martínez-Pérez G. La bisexualité au Sénegal: Histoires de vie et de santé dans le milieu dakarois [Internet]. Prensas de la Universidad de Zaragoza; 2020. Available from: https://puz.unizar.es/2395-la-bisexualite-au-senegal-histoires-de-vie-et-de-sante-dans-le-milieu-dakarois.html

[16] Gordon M. Manual of nursing diagnosis: Including all diagnostic categories approved by the North American Nursing Diagnosis Association. 12th ed. Jones and Bartlett; 2010.

[17] Spitzer RL, Kroenke K, Williams JBW, Löwe B. A brief measure for assessing generalized anxiety disorder: the gad-7. Arch Intern Med [Internet]. 2006 May 22 [cited 2022 Oct 16];166(10):1092. Available from: http://archinte.jamanetwork.com/article.aspx?doi=10.1001/archinte.166.10.1092 16717171 10.1001/archinte.166.10.1092

[18] Kroenke K, Spitzer RL. The phq-9: a new depression diagnostic and severity measure. Psychiatric Annals [Internet]. 2002 Sep [cited 2022 Oct 16];32(9):509–15. Available from: https://journals.healio.com/doi/10.3928/0048-5713-20020901-06

[19] Wahlert L, Fiester A. Queer bioethics: why its time has come: editorial. Bioethics [Internet]. 2012 Jan [cited 2022 Oct 16];26(1):ii–iv. Available from: https://onlinelibrary.wiley.com/doi/10.1111/j.1467-8519.2011.01957.x 10.1111/j.1467-8519.2011.01957.x 22168442

[20] Leininger M. Towards conceptualization of transcultural health care systems: concepts and a model. J Transcult Nurs [Internet]. 1993 Jan [cited 2022 Oct 16];4(2):32–40. Available from: http://journals.sagepub.com/doi/10.1177/104365969300400206 8507434 10.1177/104365969300400206

[21] Wahlert L, Fiester A. Repaving the road of good intentions: *LGBT health care and the queer bioethical lens*. Hastings Center Report [Internet]. 2014 Sep [cited 2022 Oct 16];44(s4):S56–65. Available from: https://onlinelibrary.wiley.com/doi/10.1002/hast.373 25231790 10.1002/hast.373

[22] Wright LW Jr, Adams HE, Bernat J. Development and Validation of the Homophobia Scale. Journal of Psychopathology and Behavioral Assessment [Internet]. 1999 [cited 2022 Oct 16];21(4):337–47. Available from: http://link.springer.com/10.1023/A:1022172816258

[23] Van der Elst EM, Smith AD, Gichuru E, Wahome E, Musyoki H, Muraguri N, et al. Men who have sex with men sensitivity training reduces homoprejudice and increases knowledge among Kenyan healthcare providers in coastal Kenya. Journal of the International AIDS Society. 2013 Dec;16:18748. doi: 10.7448/IAS.16.4.18748 24321111 PMC3852129

[24] Mapayi BM, Oginni OO, Akinsulore A, Aloba OO. Homophobia and perceptions about homosexuality among students of a tertiary institution in Nigeria. Gender and Behaviour. 2016 Dec 1;14(3):7624–37.

[25] Van der Elst EM, Smith AD, Gichuru E, Wahome E, Musyoki H, Muraguri N, et al. MSM Sensitivity Training Reduces Homoprejudice and Increases Knowledge among Kenyan Health Care Providers in Coastal Kenya. Strengthening HIV health care services for men who have sex with men in coastal Kenya. 2013;16:63. 10.7448/IAS.16.4.18748 PMC3852129 24321111

[26] Tucker A, Liht J, de Swardt G, Arendse C, McIntyre J, Struthers H. Efficacy of tailored clinic trainings to improve knowledge of men who have sex with men health needs and reduce homoprejudicial attitudes in South Africa. LGBT health. 2016 Dec 1;3(6):443–50. doi: 10.1089/lgbt.2016.0055 27835058

[27] Traicoff DA, Basarab D, Ehrhardt DT, Brown S, Celaya M, Jarvis D, et al. Using predictive evaluation to design, evaluate, and improve training for polio volunteers. Pedagogy in Health Promotion [Internet]. 2018 Mar [cited 2022 Oct 16];4(1):35–42. Available from: http://journals.sagepub.com/doi/10.1177/2373379917739012 29457126 10.1177/2373379917739012 PMC5812023

[28] Douglas CJ, Kalman CM, Kalman TP. Homophobia among physicians and nurses: an empirical study. PS [Internet]. 1985 Dec [cited 2022 Oct 16];36(12):1309–11. Available from: http://psychiatryonline.org/doi/abs/10.1176/ps.36.12.1309 4086006 10.1176/ps.36.12.1309

[29] lackwell CW, Kiehl EM. Homophobia in registered nurses: impact on LGB youth. Journal of LGBT Youth [Internet]. 2008 Sep 24 [cited 2022 Oct 16];5(4):28–48. Available from: http://www.tandfonline.com/doi/abs/10.1080/19361650802222989

[30] McNamara G, Joyce P, O’Hara J. Evaluation of adult education and training programs. In: International Encyclopedia of Education [Internet]. Elsevier; 2010 [cited 2022 Oct 16]. p. 548–54. Available from: https://linkinghub.elsevier.com/retrieve/pii/B978008044894701647X

[31] Scheibe AP, Duby Z, Brown B, Sanders EJ, Bekker LG. Attitude shifts and knowledge gains: Evaluating men who have sex with men sensitisation training for healthcare workers in the Western Cape, South Africa. South Afr j HIV med [Internet]. 2017 Mar 31 [cited 2022 Oct 16];18(1). Available from: https://sajhivmed.org.za/index.php/hivmed/article/view/673 doi: 10.4102/sajhivmed.v18i1.673 29568621 PMC5843261

[32] Kapanda L, Jumbe V, Izugbara C, Muula AS. Healthcare providers’ attitudes towards care for men who have sex with men (MSM) in Malawi. BMC Health Serv Res [Internet]. 2019 Dec [cited 2022 Oct 16];19(1):316. Available from: https://bmchealthservres.biomedcentral.com/articles/10.1186/s12913-019-4104-3 31101107 10.1186/s12913-019-4104-3 PMC6525370

