# Supplementary material for: The design and evaluation of a training program on culturally competent psychosocial care provision for men who have sex with men in Senegal
Source: PLoS One. 2023 Jul 11;18(7):e0288018. doi: 10.1371/journal.pone.0288018 (PMC10335694; doi:10.1371/journal.pone.0288018)
Supplement: S1 Table — (DOCX) [file pone.0288018.s001.docx]

S1 Table. Supplementary Material: Knowledge questionnaire

|  | Question | Possible answers |
| --- | --- | --- |
| 1 | Which of the following is not a characteristic of a psychosocially healthy person? | Showing awareness of one’s strengths and weaknesses |
|  |  | **Suppressing negative emotions as a form of coping with hardships** |
|  |  | Practising regular self-care |
|  |  | Self-criticism |
| 2 | Which of the following is not among the WHO Conceptual Framework of the Social Determinants of Health? | Race/ethnicity |
|  |  | **Sexual orientation** |
|  |  | Gender |
|  |  | Education |
| 3 | Data from the Northern Hemisphere shows that MSM are more likely to experience psychosocial disorders than their non-MSM counterparts. What do you think is the reason? | **MSM, especially those in homophobic environments, experience stigma and discrimination which, in turn, causes poor psychosocial health** |
|  |  | MSM are more likely to get HIV/AIDS, therefore, getting an HIV-positive diagnosis can cause one’s psychosocial health to deteriorate |
|  |  | MSM are less likely to engage in meaningful romantic relationships and are generally promiscuous, a trait that does not favour good psychosocial health |
|  |  | MSM are generally less religious, and therefore, suffer from psychosocial disorders as a result |
| 4 | What is the correct term to refer to this statement “One’s gender must correspond to one’s biological sex. The only natural and acceptable form of sexual or romantic attraction is between these two predefined opposite genders (women and men)”? | Heterosexuality |
|  |  | Homosexuality |
|  |  | Gender nonconformity |
|  |  | **Heteronormativity** |
| 5 | Which of the following is true about the receptive MSM partner (also known as “bottom”, “passive”, or “ubbi” in Senegal)? | He identifies as a woman |
|  |  | He identifies as a man |
|  |  | He identifies as a man; however, their behaviour is that of a woman |
|  |  | They are less masculine than the insertive partner |
|  |  | **Sexual behaviour does not necessarily correlate with gender expression or identity** |
| 6 | Which of the following is an example of culturally congruent care for MSM(/W)? | Referring to MSM(/W) patients as “homosexual” |
|  |  | **Evaluating the impact of spirituality and/ or religion on the experiences of your MSM(/W) clients** |
|  |  | Advocating for sexual orientation change therapy |
|  |  | Encouraging your MSM(/W) client to disclose their sexual orientation/practices to his friends and family |
| 7 | What is the first step of the psychosocial evaluation process according to the MDPHS model? | Design and implementation of a psychosocial care plan |
|  |  | Effective communication |
|  |  | **Client-provider partnership** |
|  |  | Identification of psychosocial needs |
| 8 | Which of the following is an assessment of the “Self-perception – self-concept pattern” in Marjory Gordon's Functional Health Patterns? | Do you feel like you have enough energy throughout the day to carry out your desired activities? |
|  |  | **How do you feel about your body in its current state?** |
|  |  | Do you have people who you consider to be your close friends? |
|  |  | How do you usually deal with challenges and adversity in life? |
| 9 | Which of the following tools can be used to diagnose depression? | The Generalized Anxiety Disorder scale |
|  |  | **The Patient Health Questionnaire** |
|  |  | Columbia-Suicide Severity Rating Scale |
|  |  | MacArthur Foundation Initiative on Depression and Primary Care |
| 10 | Which of the following techniques is helpful in managing moderate alcohol use disorder? | Scheduling alcohol intake to specific days each week |
|  |  | **Replacing alcoholic drinks with alcohol-free versions** |
|  |  | Identifying and avoiding triggers that cause alcohol consumption (e.g. stressful situations, being around “drinking buddies”, etc.) |
|  |  | Attending alcohol use disorder support group meetings |
| 11 | During the assessment of your client’s sexual wellbeing, the question “On a scale of 1 to 10, of all the things you did with your sexual partner(s), how many did you specifically agree to?” refers to which of the following dimensions? | Safety |
|  |  | Confidence |
|  |  | Communication and negotiation |
|  |  | **Consent** |
| 12 | Mamadou had recently come out to his family and friends as MSM. Most of them were supportive, however, a few decided to cut ties with him. However, he keeps telling himself “No one accepts me for who I am, I have no one”. Which of the following cognitive distortions is he not experiencing? | **Heaven’s reward fallacy** |
|  |  | Mental filtering |
|  |  | Emotional reasoning |
|  |  | Catastrophizing |
